# An analysis of risk factors associated with recurrent wheezing in the pediatric population

**DOI:** 10.1186/s13052-023-01437-4

**Published:** 2023-03-16

**Authors:** Yibing Zhu, Lumin Chen, Yecheng Miao, Jinying Chen, Meng Bai, Haiyan Gao, Zhirong Zhu, Yuxuan Zhang, Jianzhong Zhang, Hafiz Khuram Raza, Guanghua Liu

**Affiliations:** 1 Division of birth cohort study, Fujian maternity and children health hospital, Fuzhou, China; 2 Fujian Children’s Hospital (Fujian Branch of Shanghai Children’s Medical Center), College of Clinical Medicine for Obstetrics & Gynecology and Pediatrics, Fujian Medical University, Fuzhou, China; 3 Ningxia Medical University, Yinchuan, Ningxia China; 4 Department of Computer Technology, Fujian Obstetrics and Gynecology Hospital, Fuzhou, China; 5 Division of birth cohort study, Fujian obstetrics and gynecology hospital, Fuzhou, China; 6 Department of information center, Fujian children hospital, Fuzhou, China; 7 Department of clinical medicine, China medical university, Shenyang, China; 8 Department of clinical research, Unimed Scientific Inc, Wuxi, China; 9 Fujian Maternity and Child Health Hospital, Fuzhou, China; 10 Fujian Maternity and Child Health Hospital, Hengyu Road 966, Gushan Town, Jin’an District, Fuzhou City, Fujian Province China

**Keywords:** Recurrent wheezing, Risk factor, Wheezing, Asthma, PICU

## Abstract

**Background:**

Recurrent wheezing is a common clinical problem in early childhood, which is associated with significant morbidity. There is no international consensus on the management and prevention of recurrent wheezing; therefore, identifying the risk factors associated with recurrent wheezing is crucial to prevent episodes of wheezing in young children.

**Methods:**

In this retrospective study, we collected the data of 24,737 patients who were admitted to our hospital between 27th April 2012 and 11th September 2019. After screening for patients with wheezing, we identified 8572 patients with a primary diagnosis of pneumonia with wheezing. Patients’ clinical data were collected from the hospital medical records. Patients were stratified for age in the groups of < 6 months, 6–12 months, and > 12 months.

**Results:**

Among the 8569 pediatric pneumonia patients with wheezing, there were 343 patients with recurrent wheezing. Most enrolled patients were under 6 months of age (45.17%) and had a normal birth weight (86.95%). Winter was the most common onset season for the first episode of wheezing, while spring was the most common season for the second episode of wheezing for those with recurrent wheezing. The univariate and multivariate logistic regression analysis for the risk factor associated with recurrent wheezing showed that male gender, past history of respiratory and cardiovascular diseases, low birth weight, development of severe pneumonia, and PICU admission were significantly associated with recurrent wheezing.

**Conclusion:**

Male gender, past history of respiratory and cardiovascular diseases, low birth weight, severe pneumonia, and PICU admission are independent risk factors of recurrent wheezing in the pediatric population.

## Background

Wheezing is characterized by a continuous high-pitched expiratory sound due to turbulence caused by reduced airway caliber [1]. Recurrent wheezing, defined as more than one episode of wheezing in one year, is a common clinical problem in young children. Epidemiological studies have suggested that about 50% of children suffer from at least one wheezing episode in the first six years of their lives [2]. The most common cause of recurrent wheezing is asthma [3], while children can also develop several episodes of wheezing due to recurrent viral infection of the respiratory tract [4, 5]. Other causes include gastroesophageal reflux disease, bronchiolitis, cystic fibrosis, vocal cord abnormality, cardiac disorders, airway structural abnormalities, and foreign body aspiration [3]. Wheezing episodes severely impact children’s quality of life and are common causes of emergency department visits and hospitalization in early childhood [6]. A study by Bisgaard et al. [2] has reported that the rate of presentation to the emergency department and hospitalization due to recurrent wheezing in children aged 1–5 years was 16% and 12%, respectively. Recurrent wheezing is accompanied by other symptoms, such as cough and dyspnea, and significant morbidity. It can be challenging to treat persistent wheezing. For proper management and prevention of recurrent wheezing, it is crucial to study the risk factors associated with recurrent wheezing in children. Previous studies have sought to explore the risk factors of recurrent wheezing [7–11]; however, the data was limited and the sample size was very small. This study analyzed the clinical data of patients who were hospitalized at least twice with a primary diagnosis of pneumonia with wheezing in the past seven years. We used univariate and multivariate logistic regression analysis to identify the significance of risk factors associated with recurrent wheezing in pediatric patients.

## Methods

### Ethical statement

The study was approved by the ethics committee of Fujian Maternity and Child Health Hospital. This study has been registered in China, with the registration number being ChiCTR2000033019.

We confirm that all methods were performed in accordance with the ethical standards as laid down in the Declaration of Helsinki and its later amendments or comparable ethical standards.

### Study design

This is a retrospective study which analyzed the clinical data of patients from the hospital database.

### Study population

This study collected the data of 24,737 patients who were admitted to Fujian Maternity and Child Health Hospital between 27th April 2012 and 11th September 2019. After applying the following inclusion and exclusion criteria, patients with a primary diagnosis of pneumonia with wheezing were included in the final analysis.

Inclusion criteria:

1. Patients with a primary diagnosis of pneumonia with wheezing.
2. Patients aged 0–14 years.

Exclusion criteria:

1. Patients without pneumonia or bronchopneumonia.
2. Patients with a hospitalization length of less than 1 day or more than 2350 days.
3. Patients with a gestational length of less than 20 weeks.
4. Patients without wheezing.

### Data collection and grouping

Patients’ clinical data were collected from the hospital medical records, which included age, gender, residency location, birth weight, gestational length, feeding method, history of allergy, past history, presence of severe pneumonia, onset season, and admission to pediatric intensive care unit (PICU). Patients were stratified for age in the groups of < 6 months, 6–12 months, and > 12 months.

### Statistical analysis

Analyses were conducted using the SAS System version 9.4 (SAS Institute, Cary, NC). The baseline characteristics examined were based on the age groups and are described as proportions for categorical variables and as means, standard deviations, medians, interquartile ranges, and ranges for continuous variables. The significance of differences was assessed using the χ2 test (categorical variable), student’s t-test and Mann-Whitney U test (continuous variable) depending on the data distributions and variances. Statistical significance was set at P < 0.05. Univariate logistic regression analysis was carried out to calculate ORs and determine the risk factors for recurrence. Risk factors were considered for inclusion in the multivariate logistic regression if the P values of the univariate analysis were less than 0.05. The patterns of recurrence were demonstrated for factors including the age of first wheezing, birthweight, gestational age and season of occurrence.

## Results

### Case-screening of pneumonia patients with wheezing

**Fig. 1:**
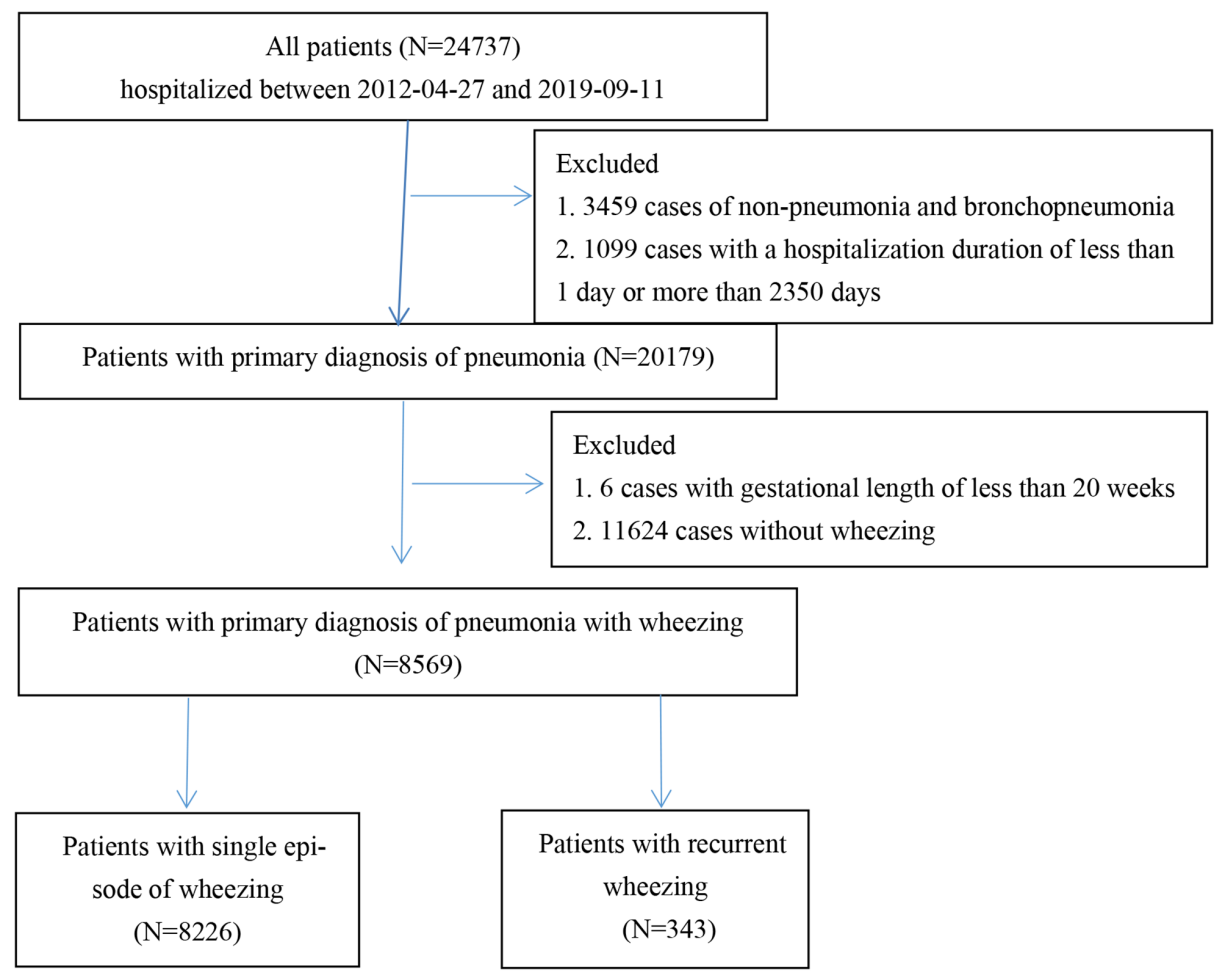
Case-screening flowchart

We initially screened 24,737 cases who were admitted to our hospital between 2012-04-27 and 2019-09-11. After excluding 3459 cases without pneumonia and bronchopneumonia, 1099 cases with a hospitalization duration of less than 1 day or more than 2350 days, 6 cases with a gestational length of less than 20 weeks, and 11,624 cases without wheezing, 8569 pediatric pneumonia patients with wheezing were enrolled in the final analysis. Among them, there were 343 patients with recurrent wheezing. Figure 1 shows the case-screening flowchart in detail.

### Age stratification of the enrolled patients

**Fig. 2:**
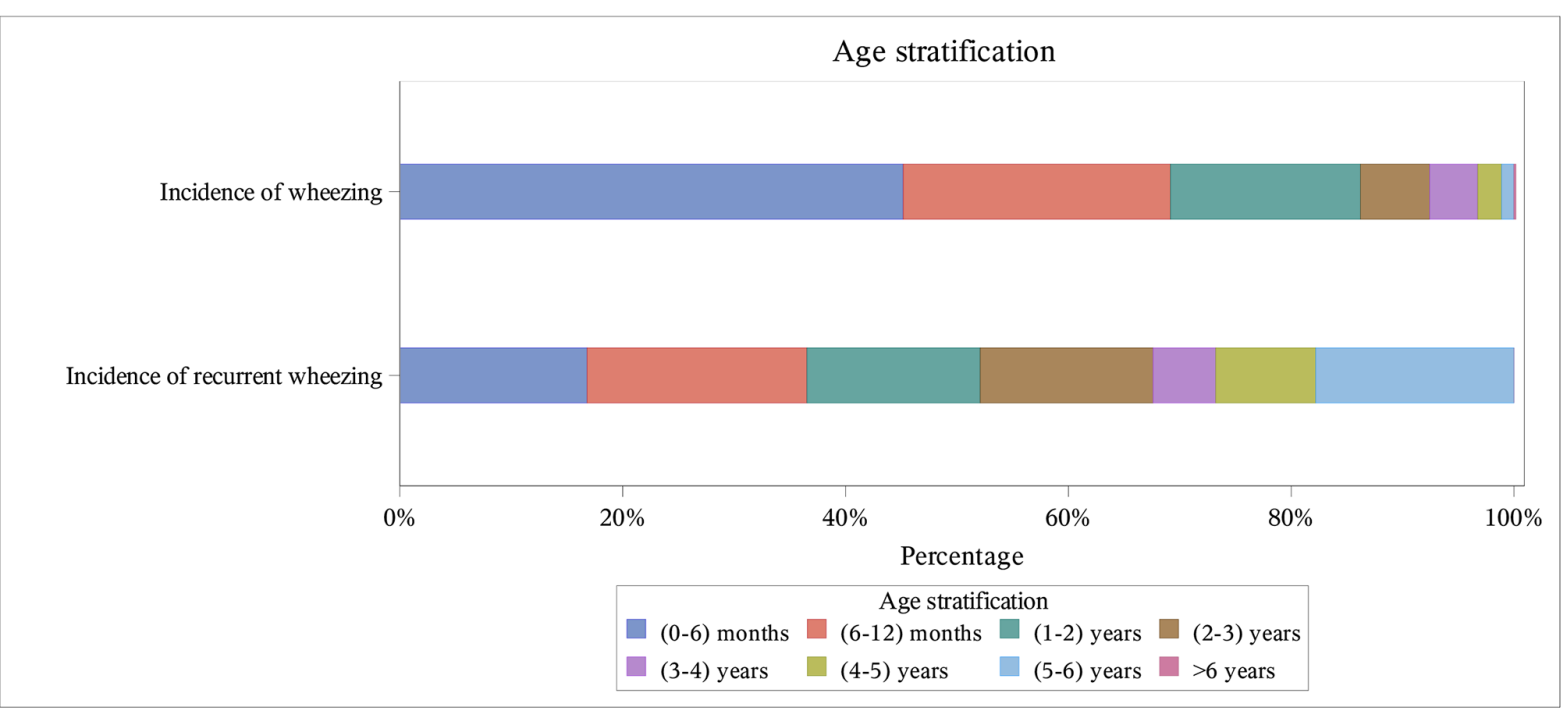
Age stratification

Most enrolled patients were under 6 months (45.17%) and 6–12 months (23.99%) of age. Similarly, the incidence of recurrent wheezing was the highest in patients under 6 months (45.77%) and 6–12 months (28.57%) of age (Table 1) (Fig. 2).

**Table 1:** The age stratification of the enrolled patients

	Statistics	Overall	Patients with recurrent wheezing
**Age stratification**	N (Missing)	8569 (0)	343 (0)
0–6 months	N (%)	3871 (45.17%)	157 (4.06%)
6–12 months	N (%)	2056 (23.99%)	98 (4.77%)
1–2 years	N (%)	1462 (17.06%)	55 (3.76%)
2–3 years	N (%)	533 (6.22%)	20 (3.75%)
3–4 years	N (%)	369 (4.31%)	5 (1.36%)
4–5 years	N (%)	184 (2.15%)	4 (2.17%)
5–6 years	N (%)	93 (1.09%)	4 (4.30%)
> 6 years	N (%)	1 (0.01%)	0 (0.00%)

### Birth history of the enrolled patients

**Fig. 3:**
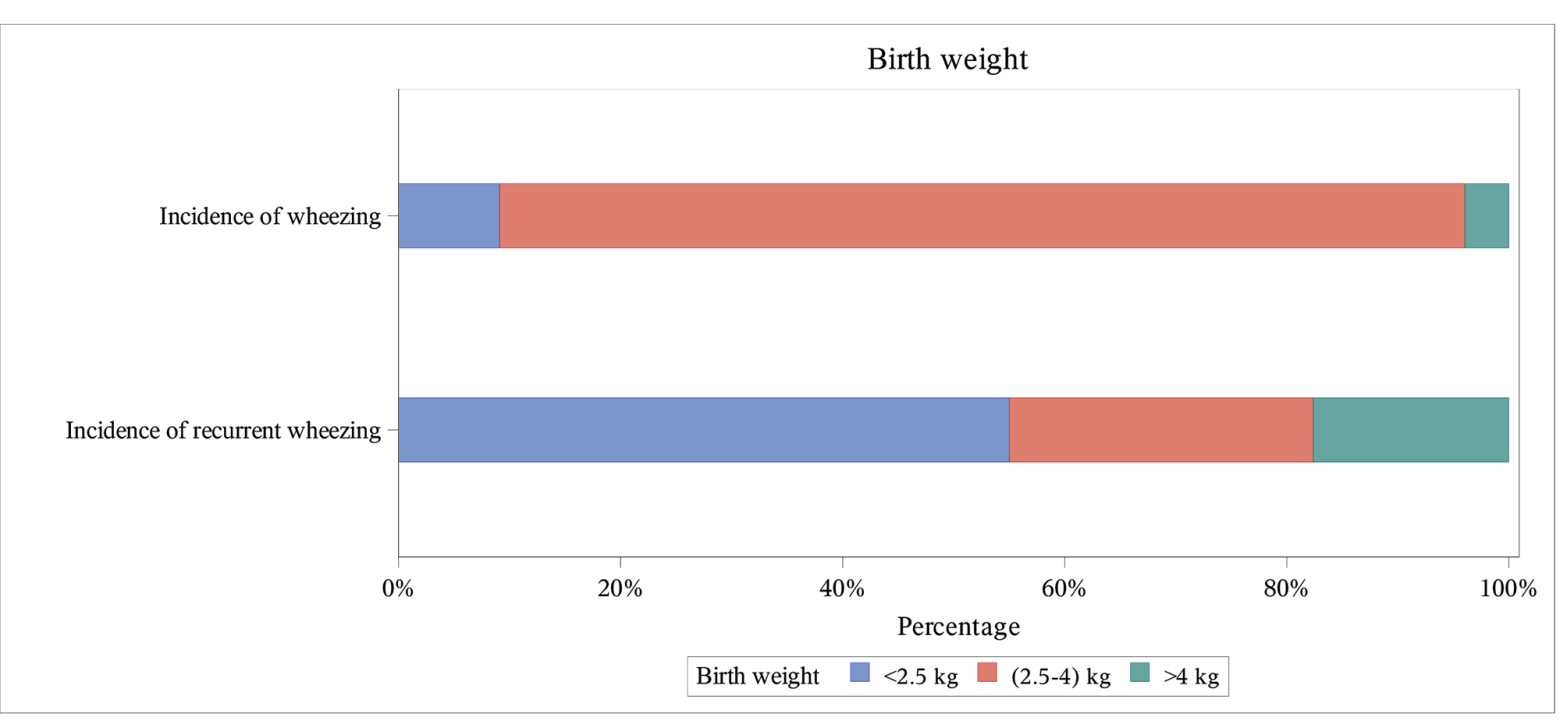
Birth weight stratification

Most patients had a normal birth weight (86.95%) and a gestational length of ≥ 37 weeks (90.26%). Similarly, the highest proportion of patients with recurrent wheezing had a normal birth weight (80.65%) and a gestational length of ≥ 37 weeks (84.94%) (Table 2) (Fig. 3). **Onset season for wheezing**:

**Table 2:** Birth weight and gestational length of the enrolled patients

	Statistics	Overall	Patients with recurrent wheezing
**Birth weight**	N (Missing)	8484 (85)	341 (2)
< 2.5 kg	N (%)	773 (9.11%)	58 (7.50%)
2.5-4 kg	N (%)	7377 (86.95%)	275 (3.73%)
> 4 kg	N (%)	334 (3.94%)	8 (2.40%)
**Gestational length**	N (Missing)	8494 (75)	332 (11)
< 34 weeks	N (%)	676 (7.96%)	44 (6.51%)
34–37 weeks	N (%)	151 (1.78%)	6 (3.97%)
≥ 37 weeks	N (%)	7667 (90.26%)	282 (3.68%)

Winter was the most common onset season for the first episode of wheezing (30.34%), followed by spring (27.94%) and autumn (23.06%). Among patients with recurrent wheezing, the most common onset season for the second episode was spring (34.99%), followed by autumn (25.66%) (Table 3).

**Table 3:** Onset seasons for first and second episodes of wheezing

Onset season for first episode	Overall	Onset season for second episode			
		Spring	Summer	Autumn	Winter
Spring	2394 (27.94%)	18 (0.75%)	25 (1.04%)	33 (1.38%)	13 (0.54%)
Summer	1600 (18.67%)	8 (0.50%)	8 (0.50%)	32 (2.00%)	13 (0.81%)
Autumn	1975 (23.05%)	28 (1.42%)	5 (0.25%)	12 (0.61%)	29 (1.47%)
Winter	2600 (30.34%)	66 (2.54%)	20 (0.77%)	11 (0.42%)	22 (0.85%)

### Demographic and clinical features of enrolled patients

Patients with recurrent wheezing were stratified in the following age groups for analysis: 0–6 months, 6–12 months, and > 12 months. There were 157 patients in the 0–6 months group, 98 patients in the 6–12 months group, and 88 patients in the > 12 months group. Overall, there were more males than females, with a male-to-female ratio of 4:1. The similar difference was observed in all the groups (p = 0.1192). Most patients in all groups were urban residents (60.29% vs. 39.71%, p = 0.0766). Breastfeeding was the most common feeding method for patients in all groups (47.08%, p = 0.0117). Most patients were born full-term (83.19%, p = 0.259), with a major proportion of cases having a gestational length of more than 37 weeks (84.94%, p = 0.289). The median birth weight was 3.15 kg. The incidence of severe pneumonia was the highest for patients in the 0–6 months group (38.22%, p = 0.0004). Similarly, patients in the 0–6 months group also had the highest incidence of PICU admission (25.48%, p = 0.0098). Table 4 shows in detail the demographic and clinical features of patients with recurrent wheezing.

**Table 4:** Demographic and clinical features of patients with recurrent wheezing

	Total (N = 343)	0–6 months (N = 157)	6–12 months (N = 98)	> 12 months (N = 88)	P-value
Gender					
Male	275 (80.17%)	131 (83.44%)	80 (81.63%)	64 (72.73%)	0.1192
Female	68 (19.83%)	26 (16.56%)	18 (18.37%)	24 (27.27%)	
**Residency**					
Urban	205 (60.29%)	88 (56.41%)	55 (57.29%)	62 (70.45%)	0.0766
Rural	135 (39.71%)	68 (43.59%)	41 (42.71%)	26 (29.55%)	
**Onset season**					
Spring	89 (25.95%)	48 (30.57%)	22 (22.45%)	19 (21.59%)	0.2238
Summer	61 (17.78%)	25 (15.92%)	15 (15.31%)	21 (23.86%)	
Autumn	74 (21.57%)	29 (18.47%)	22 (22.45%)	23 (26.14%)	
Winter	119 (34.69%)	55 (35.03%)	39 (39.80%)	25 (28.41%)	
**Feeding method**					
Breast feeding	161 (47.08%)	62 (39.49%)	46 (47.42%)	53 (60.23%)	0.0117
Mixed feeding	90 (26.32%)	50 (31.85%)	20 (20.62%)	20 (22.73%)	
Artificial feeding	91 (26.61%)	45 (28.66%)	31 (31.96%)	15 (17.05%)	
**Full term delivery**					
Yes	282 (83.19%)	128 (82.05%)	76 (80.00%)	78 (88.64%)	0.259
No	57 (16.81%)	28 (17.95%)	19 (20.00%)	10 (11.36%)	
**Gestational length**					
< 34 weeks	44 (13.25%)	21 (13.91%)	16 (17.20%)	7 (7.95%)	0.289
34–37 weeks	6 (1.81%)	2 (1.32%)	1 (1.08%)	3 (3.41%)	
37 ≥ weeks	282 (84.94%)	128 (84.77%)	76 (81.72%)	78 (88.64%)	
**Mode of delivery**					
Vaginal delivery	229 (68.15%)	105 (68.63%)	64 (65.31%)	60 (68.18%)	0.9324
Cesarean section	107 (31.85%)	48 (31.37%)	32 (32.65%)	27 (30.68%)	
**Birth weight stratification**	3.15 (2.75–3.50)	3.1 (2.75–3.45)	3.1 (2.60–3.40)	3.2 (2.94–3.55)	0.0974
< 2.5 kg	58 (17.01%)	27 (17.42%)	23 (23.47%)	8 (9.09%)	0.093
2.5-4 kg	275 (80.65%)	123 (79.35%)	74 (75.51%)	78 (88.64%)	
> 4 kg	8 (2.35%)	5 (3.23%)	1 (1.02%)	2 (2.27%)	
**History of allergies**					
Yes	30 (8.75%)	6 (3.82%)	10 (10.20%)	14 (15.91%)	0.0048
No	313 (91.25%)	151 (96.18%)	88 (89.80%)	74 (84.09%)	
**Past history**					
Yes	102 (29.74%)	37 (23.57%)	36 (36.73%)	29 (32.95%)	0.061
No	241 (70.26%)	120 (76.43%)	62 (63.27%)	59 (67.05%)	
**Past history classification**	102	37	36	29	
Non-neonatal respiratory system diseases	80 (78.43%)	25 (67.57%)	29 (80.56%)	26 (89.66%)	
Non-neonatal cardiovascular diseases	41 (40.20%)	19 (51.35%)	12 (33.33%)	10 (34.48%)	
Neonatal respiratory diseases	27 (26.47%)	14 (37.84%)	10 (27.78%)	3 (10.34%)	
**C-reactive protein**					
Negative	139 (78.53%)	72 (88.89%)	29 (63.04%)	38 (76.00%)	0.0026
Positive	38 (21.47%)	9 (11.11%)	17 (36.96%)	12 (24.00%)	
**Severe pneumonia**					
Yes	96 (27.99%)	60 (38.22%)	17 (17.35%)	19 (21.59%)	0.0004
No	247 (72.01%)	97 (61.78%)	81 (82.65%)	69 (78.41%)	
**PICU admission**					
Yes	64 (18.66%)	40 (25.48%)	11 (11.22%)	13 (14.77%)	0.0098
No	279 (81.34%)	117 (74.52%)	87 (88.78%)	75 (85.23%)	

### Univariate and multivariate logistic regression analysis

The univariate and multivariate logistic regression analysis showed that risk factors significantly associated with recurrent wheezing were male gender (OR, 1.74; 95% CI, 1.33–2.28; p < 0.0001 and OR, 1.81; 95%CI, 1.38–2.38; p < 0.0001), past history of respiratory and cardiovascular diseases (OR, 1.84; 95% CI, 1.45–2.33, p < 0.0001 and OR, 1.51; 95% CI, 1.18–1.94; p = 0.0011), low birth weight (OR, 2.01; 95% CI, 1.53–2.63, p < 0.0001 and OR, 1.70; 95% CI, 1.29–2.24, p = 0.0002), development of severe pneumonia (OR, 2.17; 95% CI, 1.70–2.76, p < 0.0001 and OR, 2.90; 95% CI, 1.96–4.30, p < 0.0001), and PICU admission (OR, 1.69, 95% CI, 1.28–2.24, p = 0.0002 and OR, 0.56, 95% CI, 0.36–0.87, p = 0.01) (Tables 5 and 6).

**Table 5:** Univariate logistic regression analysis of the risk factors associated with recurrent wheezing

Variables	Wald Chi Square statistics	P-value	95% CI for adjust OR
Gender	16.1613	< 0.0001	1.74 [1.33–2.28]
Age	1.6088	0.2047	0.92 [0.81–1.05]
Gestational length	0.4691	0.4934	0.85 [0.54–1.35]
Residency	1.9941	0.1579	1.17 [0.94–1.46]
Feeding method	4.9673	0.0258	1.17 [1.02–1.33]
Onset season	2.1476	0.1428	1.07 [0.98–1.17]
Past history of cardiovascular and respiratory system diseases	25.028	< 0.0001	1.84 [1.45–2.33]
Full term delivery	17.657	< 0.0001	0.53 [0.40–0.71]
Birth weight (kg)	25.3034	< 0.0001	2.01 [1.53–2.63]
Parity	11.0022	0.0009	1.32 [1.12–1.56]
Gravidity	15.4919	< 0.0001	1.36 [1.17–1.58]
Severe pneumonia	38.8297	< 0.0001	2.17 [1.70–2.76]
Oxygen administration	5.4544	0.0195	1.31 [1.04–1.64]
PICU admission	13.6456	0.0002	1.69 [1.28–2.24]
C-reactive protein	1.5526	0.2127	1.01 [1.00-1.01]

**Table 6:** Multivariate logistic regression analysis

Variables	Wald Chi Squares statistics	P-value	95% CI for adjust OR
Gender	18.1085	< 0.0001	1.81 [1.38–2.38]
Onset season	2.3644	0.1241	1.08 [0.98–1.18]
Past history of cardiovascular and respiratory system diseases	10.6122	0.0011	1.51 [1.18–1.94]
Birth weight (kg)	14.3581	0.0002	1.70 [1.29–2.24]
Severe pneumonia	28.3067	< 0.0001	2.90 [1.96–4.30]
PICU admission	6.6268	0.01	0.56 [0.36–0.87]

## Discussion

Wheezing is fairly common in children, with about 50% of all children suffering from at least one wheezing episode during the first six years of their lives [12]. Children who have the first wheezing episode early in life usually tend to continue having wheezing episodes till six years of age [4]. Recurrent wheezing is associated with frequent hospital and emergency department visits. Moreover, patients with recurrent wheezing are at increased risk of developing acute wheezing episodes, asthma, and respiratory tract infections [13]. We found that the incidence of wheezing and recurrent wheezing was the highest in patients < 1 year of age and winter was the most common season for the first episode of wheezing, while spring was the most common season for the second episode of wheezing. This study classified the enrolled patients into three categories based on their age: 0–6 months, 6–12 months, and > 12 months. Our findings showed that males predominated females, most patients were urban residents, and breastfeeding was the most common feeding method. Male gender, past history of respiratory and cardiovascular diseases, low birth weight, severe pneumonia, and PICU admission are independent risk factors of recurrent wheezing in the pediatric population. The results of univariate and multivariate logistic regression analysis showed that male gender, past history of respiratory and cardiovascular diseases, low birth weight, severe pneumonia, and PICU admission were independent risk factors of recurrent wheezing.

Recurrent wheezing is more common in patients who experience the first episode of wheezing at an early age. Moreno-Galdó et al. [14] found that the rates of wheezing were similar during the first two years of life and declined later. This study found that patients under 6 months of age and 6–12 months of age had the highest incidence of recurrent wheezing (45.8% and 28.6%, respectively), which is consistent with the reports of previous studies. In this study, we found that winter (30.34%) and Spring (27.94%) were the most common onset seasons for the first wheezing episode, while, for the second wheezing episode, winter was the most common onset season. Similar results have also been reported in the literature. The association between respiratory syncytial virus (RSV) infection in early life and recurrent wheezing has been reported in the literature [15]. A study by Obando-Pacheco et al. [16] on the global seasonality of RSV reported that the peak activity of RSV was seen in winter for most countries, and high activity of RSV was seen at the beginning of Spring in northern countries. Previous studies have found that risk factors associated with recurrent wheezing in the pediatric population are lower and upper respiratory tract infection, passive smoking, personal history of atopy, and lower gestational age [8, 17]. This study found that male gender, past history of respiratory and cardiovascular diseases, low birth weight, development of severe pneumonia, and PICU admission were significantly associated with recurrent wheezing. Multiple studies have shown that males are more likely to develop recurrent wheezing than females [8, 9, 18]. Furthermore, a study has reported that the onset of asthma and recurrent wheezing is earlier in males compared to females [12]. The airways are narrower in male infants compared to females, and air pollution coupled with anatomically disadvantaged airways can lead to a higher incidence of recurrent wheezing [19]. This study found that there were more males with recurrent wheezing than females (80.17% vs. 19.83%), which is consistent with previous studies. The association between low birth weight and the risk of recurrent wheezing is evident and has been reported by multiple studies [8, 20–22]. Low birth weight is associated with conditions such as premature birth and intrauterine growth restriction, which can lead to compromised pulmonary development and poor respiratory functions, thereby increasing the risk of recurrent wheezing. Measures to correct maternal and fetal risk factors to avoid low birth weight in children can decrease the risk of recurrent wheezing. Similarly, appropriate and timely management of respiratory and cardiac disorders, especially preventing respiratory tract infection and severe pneumonia, can reduce the risk of recurrent wheezing in later life.

This study has some limitations. First, there is potential bias due to the retrospective nature of the study. Second, this is a single-center study. Further multicenter studies with large sample sizes are required to confirm our findings. Third, this study only enrolled patients who were hospitalized twice due to pneumonia with wheezing, and therefore, the record of further wheezing episodes was not available.

## Conclusion

Male gender, past history of respiratory and cardiovascular diseases, low birth weight, severe pneumonia, and PICU admission are independent risk factors of recurrent wheezing in the pediatric population.

## Data Availability

The data will be available on request to the corresponding author.
